# Comparative Proteome Analysis of Cryopreserved Flagella and Head Plasma Membrane Proteins from Sea Bream Spermatozoa: Effect of Antifreeze Proteins

**DOI:** 10.1371/journal.pone.0099992

**Published:** 2014-06-18

**Authors:** Loredana Zilli, José Beirão, Roberta Schiavone, Maria Paz Herraez, Antonio Gnoni, Sebastiano Vilella

**Affiliations:** 1 Department of Biological and Environmental Sciences and Technologies, University of Salento, Lecce, Italy; 2 Department of Molecular Biology, University of León, León, Spain; 3 Department of Basic Medical Sciences, Neurosciences and Sense Organs, University of Bari, Bari, Italy; Universidad Nacional Autónoma de México, Mexico

## Abstract

Cryopreservation induces injuries to fish spermatozoa that in turn affect sperm quality in terms of fertilization ability, motility, DNA and protein integrity and larval survival. To reduce the loss of sperm quality due to freezing-thawing, it is necessary to improve these procedures. In the present study we investigated the ability of two antifreeze proteins (AFPI and AFPIII) to reduce the loss of quality of sea bream spermatozoa due to cryopreservation. To do so, we compared viability, motility, straight-line velocity and curvilinear velocity of fresh and (AFPs)-cryopreserved spermatozoa. AFPIII addition to cryopreservation medium improved viability, motility and straight-line velocity with respect to DMSO or DMSO plus AFPI. To clarify the molecular mechanism(s) underlying these findings, the protein profile of two different cryopreserved sperm domains, flagella and head plasma membranes, was analysed. The protein profiles differed between fresh and frozen-thawed semen and results of the image analysis demonstrated that, after cryopreservation, out of 270 proteins 12 were decreased and 7 were increased in isolated flagella, and out of 150 proteins 6 showed a significant decrease and 4 showed a significant increase in head membranes. Mass spectrometry analysis identified 6 proteins (4 from isolated flagella and 2 present both in flagella and head plasma membranes) within the protein spots affected by the freezing-thawing procedure. 3 out of 4 proteins from isolated flagella were involved in the sperm bioenergetic system. Our results indicate that the ability of AFPIII to protect sea bream sperm quality can be, at least in part, ascribed to reducing changes in the sperm protein profile occurring during the freezing-thawing procedure. Our results clearly demonstrated that AFPIII addition to cryopreservation medium improved the protection against freezing respect to DMSO or DMSO plus AFPI. In addition we propose specific proteins of spermatozoa as markers related to the procedures of fish sperm cryopreservation.

## Introduction

Fish sperm cryobanks are considered a useful tool for genetic management and artificial reproduction [1], [2]. For this purpose it is necessary to preserve only high quality gametes and to develop freezing methods, which allows the maintenance of the characteristics of spermatozoa. Although cryopreservation protocols of sea bream spermatozoa have been well established [3], [4], some degradation of sperm quality compared to fresh sperm are still reported [4], [5]. The freezing–thawing procedure affects DNA integrity [4], [6]–[8], membrane lipids [5], [9], [10], sperm motility [11]–[14], fertilization ability [1], [15], [16] and larval survival [17].

Antifreeze proteins (AFPs) are present in the body fluid of cold water marine fish and some terrestrial invertebrates [18]–[20]. AFPs are a class of peptides that reversibly inhibit ice crystal growth through a non-colligative mechanism [21]–[23]. Three types are commercially available: namely, AFP types I and III, and antifreeze glycoproteins (AFGP). AFPs have a dual effect in low-temperature storage: inducing ice nucleation and inhibiting re-crystallization. In the absence of the aggregation effect, AFPs act as re-crystallization inhibitors and may mitigate cryoinjury; on the other hand, when aggregation occurs, the AFPs serve as ice nucleators and lead to cell membrane damage [24]. AFPs have been applied during low-temperature preservation in different cell types [25], [26]. AFPs can also interact with plasma membrane at low-temperatures, as demonstrated for AFPI in liposomes [27].

The ability of AFPs to protect sperm quality during the freezing-thawing procedure has been previously tested in mammals [28], [29]. Few studies have so far been carried out concerning the role of AFPs in fish sperm cryopreservation [30], [31]. Recently [5], the protective effect of AFPs on plasma membrane lipid composition of sea bream spermatozoa subjected to freezing-thawing procedure has been demonstrated. However, studies of AFPs action on protein profile of cryopreserved sperm are lacking, in spite of the fact that defects in sperm proteins may compromise sperm motility, fertilization ability and the early events after fertilization [12]. It has been demonstrated that the alteration of sperm proteins (degradation and/or change of the phosphorylation state) due to the freezing-thawing procedure may contribute to the observed changes in spermatozoa quality [12], [32]–[34].

The aim of our work was to evaluate the “protective” effect of the AFPs on sperm proteins during freezing-thawing procedure and, also, to identify specific proteins of flagella and head plasma membranes spermatozoa as potential markers related to the procedures of fish sperm cryopreservation. In particular, we analyzed by 2-DE associated with Nano-LC mass spectrometry the effect of AFPs on the pattern of sperm proteins extracted from isolated flagella and head plasma membranes. Our results show a higher ability of AFPIII, as compared to AFPI, in the protection of sperm proteins during the freezing-thawing procedure. AFPI and AFPIII differently affect the protein profile of the two investigated domains.

## Materials and Methods

All chemicals, unless otherwise stated, were purchased from Sigma-Aldrich (St. Louis, MO) and were reagent grade or higher.

### Ethics Statement

Fish handling was in accordance with the European Union Directive (EEC, 1986) for the protection of animals used for experimental and other scientific purposes. Field studies were performed at the local fish farm (MARIBRIN) located to Contrada Pandi, 72100 Brindisi (BR) – Apulia, they did not involve endangered or protected species and did not implicate the sacrifice of animals. Procedures relating to care and use animals were approved by the Ethics Committee from University of Salento in accordance with the European Union regulations.

### Fish and sperm collection

Milt collection was performed in three different samplings, during late autumn (November), early winter (December) and midwinter (January) at the local fish farm (MARIBRIN) located to Contrada Pandi, 72100 Brindisi (BR) – Apulia. The study was carried out on a sexually mature broodstock of reared gilthead sea bream (*Sparus aurata)* males. The broodstock was preserved in an indoor tank at a density of 0.6 kg/m^3^. The broodstock tank was replenished with seawater at a rate of 1 l/sec, while compressed air was provided through air stones. Glithead sea bream were given pellets daily, and fresh fish food was provided once a week. The water temperature ranged between 13.5°C and 15.5°C, and the salinity was 34.2‰. To avoid fish stress, before sample collection the fish were anesthetized in 0.1 ml/l 2-phenoxy ethanol. Sperm was collected by applying gentle abdominal pressure to extrude milt that was removed from the gonopore with a syringe. Before collection the urogenital pore was cleaned to eliminate urine, water, mucus and faeces. This procedure was performed as quickly as possible and the fish were returned to anesthetic-free water to recover and were observed for at least one hour to check their health condition. The milt was transferred to a vial and kept at 4°C for 30–60 min, until its use. In each sampling 7 or 8 sperm samples (from different fish) were collected. The first step was the measurements of the spermatozoa motility parameters and viability of fresh sperm. The six semen samples that had the best quality parameters were used for the cryopreservation procedure and for the extraction of proteins.

### Cryopreservation procedure

Sperm motility and viability were analyzed and proteins extracted for four different experimental conditions: fresh (control) and cryopreserved sperm using the three different cryoprotectants as described below. Briefly, sperm was diluted 1/6 (v/v) in the extender solution (1% NaCl plus 5% DMSO) with and without AFPI or AFPIII (1 µg/ml) and loading it in 0.5 ml French straws. Sperm cryopreservation was performed as reported previously [5]. Preliminary experiments were conducted to test the best AFPI and AFPIII (A/F Protein Canada Inc., St John’s, Canada) concentrations (Table 1).

**Table 1:** Effect of AFPI and AFPIII at three different concentrations (0.1, 1 and 10 µg/ml) on motility rate and viability before freezing (fresh sperm) and after thawing.

	Fresh (N = 3)	DMSO (N = 3)	DMSO+AFP I (N = 3)			DMSO+AFPIII (N = 3)		
AFP concentration (µg/ml)			0.1	1	10	0.1	1	10
**% Motility**	73±8^a^	24±11^b^	23±7^b^	27±9^b^	28±6^b^	38±7^c^	43±6^c^	42±8^c^
**% Vitality**	82±10^a^	33±7^b^	33±6^b^	35±4^b^	36±5^b^	47±4^d^	53±8^d^	51±6^d^

Values are expressed as means ± SD.

Within each row, values with different superscript letters indicate significant statistical differences (*P*<0.05).

### Computer Assisted Sperm Analysis (CASA)

Sperm movement was videotaped using a Nikon Alphaphot 2 microscope with an x20 negative phase objective and a Sony CCD black and white video camera (SSC-M188CE). Each sperm sample was diluted 1∶100 with non-activating medium (NAM, in mg/ml: NaCl 3.5, KCl 0.11, MgCl_2_ 1.23, CaCl_2_ 0.39, NaHCO_3_ 1.68, glucose 0.08, BSA 10, pH 7.7) and motility was initiated by addition of seawater (∼13°C) (in a dilution 1∶10 sperm:sea water). The measure of motility started after 15 seconds, the time need to spot and mix sperm and sea water on well of multitest slide (12 well, ICN, Basingstoke, UK) covered with a cover slip and video-recorded. The motility of cryopreserved spermatozoa was checked immediately after thawing (without dilution) using the above reported procedure.

Videotapes were analyzed using the Hobson Sperm Tracker and associated software (Hobson Vision Ltd, Baslow, UK). For each sperm sample, two aliquots were analyzed and for each analysis ∼150 sperm tracks (mean, 136; range, 84–180) were imported into a SPSS 15.0 software for statistical analysis. The total duration of motility was timed by stopwatch when the 95% of the sperm ceased moving. Only forward-moving sperm were judged motile, those simply vibrating or turning on their axes were considered immotile. Percentage of motile sperm (MOT), motility duration (MD), curvilinear velocity (VCL) and straight-line velocity (VSL) were measured.

### Sperm viability

Percentages of live and dead sperm cells were determined using a live-cell nucleic acid stain, SYBR-14, in combination with the conventional dead-cell nucleic acid stain, propidium iodide according to the staining protocol of the live/dead sperm viability kit (Molecular Probes, Eugene, OR). Briefly, semen samples were diluted 1∶100 in NaCl 1.1% and 5 µl of diluted (1∶50) SYBR-14 dye were added to 1 ml of the sperm suspension and incubated at 36°C for 10 min. Then, 5 µl of propidium iodide were added to 1 ml sample of diluted semen, and incubated for an additional 5 minutes. The sperm suspension was loaded on a glass slide, covered with a cover slip, and immediately observed under a fluorescent microscope equipped with appropriate filters. SYBR-14 stains the nucleus of live sperm green, while dead or membrane-damaged spermatozoa are stained red by the propidium iodide. At least 200 cells were evaluated per sample. Only sperm samples with viability >65% were used for the experiment.

### Fertilization Assays

The fertilizing ability of fresh and frozen-thawed semen was evaluated following the protocol proposed by Barbato *et al.* [35]. Triplicate batches of eggs from two females were inseminated with fresh or thawed spermatozoa obtained from three different males. Each sperm sample was divided in two aliquots one for the fertilization trials with fresh sperm and the other for the fertilization trials with spermatozoa cryopreserved in the presence of AFPIII. Total number of oocytes used in each batch was approximately 200 and a ∼260,000 spermatozoa/oocyte ratio was used to perform fertilization assay with fresh and post-thawed spermatozoa. The quality parameters (motility and viability) of the sperm samples used in the fertilization trials was comparable to that measured in the spermatozoa used for proteomic analysis (respectively, fresh and DMSO plus AFPIII cryopreserved samples).

To determine the hatching rate, the number of hatched larvae and dead eggs in a 200 ml sample was counted. The hatching rate was determined as the proportion of hatched eggs to total eggs. All fertility tests were performed in triplicate for each sperm sample.

### Head plasma membrane and flagella lsolation

Head plasma membrane and flagella isolation was obtained as previously described [5]. Sperm samples were centrifuged (4,000×g for 15 min at 4°C) to eliminate the seminal plasma, and washed with 1% NaCl (addition of five times 1% NaCl volume followed by centrifugation and elimination of the supernatant). For head and flagella separation, samples were resuspended four times their volume in 1% NaCl and passed ten times through a 50 cm×0.5 mm inner diameter capillary attached to a 20 ml syringe. Percentage of separation was controlled by observation at light microscopy.

Eight ml of the samples were carefully layered over 24 ml of a 0.5–2 M sucrose gradient, made up in 4 steps (6 ml of 0.5, 1, 1.5 and 2 M) prepared in ultracentrifugation tubes. After centrifugation at 28,000×g for 45 min at 4°C two bands were visible, an upper band (0.5 M sucrose) consisting of flagella, and lower band (between 1.5 and 2 M sucrose) containing spermatozoid heads. Using of glass Pasteur pipettes the two bands were individually collected and diluted in 1% NaCl to a total volume of 40 ml. Flagella suspensions were centrifuged at 5,000×g for 20 min at 4°C, and the pellets used for protein extraction. Head suspensions were centrifuged at 3,000×g for 20 min at 4°C and 30 ml of distilled water were added to the pellets, and the 30 ml suspensions were vortexed for 10 s. This operation allowed the spermatozoa heads to be lysed. Head suspensions were centrifuged for 25 min at 1,000×g at 4°C to sediment the cellular debris, and the supernatants containing the head plasma membranes were recovered. To concentrate the head plasma membranes, supernatants were ultracentrifuged for 20 min at 28,000×g at 4°C. The head plasma membrane pellets were used for protein extraction.

### Protein extraction

For protein extractions, samples of flagella and head plasma membranes were resuspended in the lysis buffer containing 8 M urea, 2% Chaps, and 18.6 mM dithiothreitol. After incubation for 1 h at room temperature the samples were centrifuged at 12,000 *g* for 5 min at 4°C. The supernatants were then recovered, and stored at −80°C until the electrophoretic analyses were carried out. The protein concentration was measured by the Quick Start Bradford Protein Assay (Bio-Rad) using BSA as the standard.

### Isoelectric Focusing

Isoelectric focusing (IEF) was performed on immobilized pH gradients (IPG; pH 3–10, 13 cm) with IPGphor (Amersham Biosciences). 60 µg of proteins were used for analytical runs, and 800 µg of proteins were employed for preparative runs in a total volume of 250 µl of rehydrating buffer (8 M urea, 2% Chaps, 18.6 mM dithiothreitol, and 1% IPG buffer pH 3–10 [Amersham Biosciences]). Strips were rehydrated for 12 h. Focusing was performed with 50 mA per strip for 1 h at 500 V, 1 h at 1000 V, and 2 h at 8000 V at 20°C. After IEF, the strips were equilibrated in the first step with 6 M urea, 30% glycerol, 2% SDS, 50 mM Tris pH 6.8, and 2% dithiothreitol for 15 min, and in the second step with 2.5% iodoacetamide, instead of dithiothreitol, for another 15 min. As a tracking dye, a few grains of bromophenol blue were added.

### SDS-PAGE

Separation of the second dimension was performed in 12.5% SDS/polyacrylamide gels (14×16 cm) using the Hofer SE 600 Ruby System (Amersham Biosciences). The running conditions were 15 mA/gel for 15 min and 30 mA/gel for 5 h. Once the bromophenol blue had reached the anode, the gels were fixed and stained by a standard silver staining protocol (Amersham Biosciences). Coomassie R-250 staining protocol (Roti-Blue; Roth, Karlsruhe, Germany) was used to visualize protein spots in preparative gels.

### Acquisition and Analysis of Two-dimensional Gels

The stained two-dimensional gels were scanned on ImageMaster Gel Scanner (Amersham Biosciences). The image analysis was performed using the ImageMaster 2D Elite software version 3.1 (Amersham Biosciences). Protein spots were detected using automated routines from the software combined with manual editing to remove artefacts. The system worked on a series of gels (obtained by using proteins extracted by fresh samples) by giving a special status to a particular gel (the reference gel) and comparing all of the other gels, to the reference gel. A spot number was assigned to each spot within the reference gel, and it was used in the subsequent description to refer to individual spots. Three replicates of each 2D-gel were used to perform the image analysis.

### Protein analysis by Nano-LC-MS/MS and database searching

The spots showing differences between treatments were analysed as reported in Taurino *et al*. [36] with minor modifications. Spots were excised and in-gel digested using trypsin Profile IGD Kit (Sigma-Aldrich), following manufacturer’s instructions. Proteolytic peptides were pre-concentrated on a reversed phase nano-pre-column (NanoEase trap column, dC18 Atlantis, Waters) using a CapLC micro HPLC (Waters) at 30 µl/min flow rate for 5 minutes in 0.1% Formic Acid (FA). Separation was performed on a nano-column (NanoEase Atlantis, C18, 75 µm×100 mm, 100 Å, Waters), using binary gradient (solvent A: 0.1% FA, solvent B: 0.1% FA 84% ACN) from 5 to 50% of solvent B in 60 min with a flow rate of approximately 300 nl/min. Data-directed analysis (DDA; parent survey) mass spectra were acquired on Q-TOF instrument (Q-TOF micro, Waters) equipped with a nanoflow electrospray ion source. The mass spectrometer operates in positive ion mode with a source temperature of 100°C and a voltage of 3.5 kV applied to the probe tip. The instrument was externally calibrated with a multi-point calibration based on the MS/MS fragment ions of doubly charged Fibrinopeptide-B (Sigma-Aldrich). Argon was used as collision gas. Mass spectra were acquired by the Q-TOF analyzer in the V-mode of operation and spectra were integrated over one second interval. MS and MS/MS data were acquired in continuum mode. MassLynx 4.0 (Waters) software was employed to perform MS/MS analysis on up to fourthly charged precursor ions. Data in PKL format were generated by ProteinLynx Global Server 2.2 (Waters). Database search was performed by Mascot program (http://www.matrixscience.com) using the following criteria: type of search: MS/MS Ion Search, NCBInr database (released on December 28 2013), with taxonomy: Chordata; it was assumed that mass values were monoisotopic, set as fixed modifications: carbamidomethylation of cysteine, and no variable modifications. The MS/MS Ion Search method allowed for zero-missed cleavage for tripsyn, and the peptide mass tolerance was set as 0.2 Da and the fragment mass tolerance as 0.6 Da; peptide charge was set +2, +3 and +4.

### Statistical analysis

All statistics were conducted using the software SPSS 15.0 for Windows. Sperm quality parameters (motility and viability data) were analyzed with a general linear model with Bonferroni adjustment. Percentage of motility, curvilinear velocity (VCL), straight-line velocity (VSL) and viability data were normalized through arcsine transformation. P<0.05 was considered statistically significant. Results are reported as mean values ± SD for % motility and vitality and as mean values ± SEM for VCL and VSL.

The amount of protein present in a spot was taken as the area of the spot multiplied by the density and referred to as the volume. Following removal of background, the spot volumes were normalized to the total protein detected for each gel by dividing the individual spot volume by the sum of all spot volumes and multiplying by 100. The normalized spot volume is referred to as abundance. Normalized volume of each spot was evaluated as mean ± standard deviation of spot normalized volumes measured in all gels produced with the same cryopreservation protocol. Comparison of proteins from gels obtained by using extracts from spermatozoa differently cryopreserved was assessed using Mann–Whitney test and relationships were considered statistically significant when P<0.05.

## Results

### Effect of antifreeze proteins on spermatozoa motility parameters, viability and fertilization ability

As reported in Table 2, sea bream semen cryopreserved in DMSO-containing extender showed, after thawing, a significant reduction of motility rate and duration, viability and straight-line velocity (VSL) with respect to fresh semen. The addition of AFPIII to DMSO extender improved the protection against freezing with respect to DMSO or DMSO plus AFPI. A percentage increase of motility rate and viability, and VSL was observed as compared to DMSO alone. No significant changes were found by the addition of AFPI to DMSO extender. In agreement with Beirao *et al* [5] curvilinear velocity (VCL) seems to be almost unaffected under the tested experimental conditions. Motility duration shows a significant reduction after cryopreservation procedure in all conditions.

**Table 2:** Effects of different cryoprotectants on different motility and viability parameters in sea bream spermatozoa before freezing (fresh sperm) and after different thawing conditions.

Samples	Motility Duration (sec)	% Motility	% Vitality	VSL (µm/sec)	VCL (µm/sec)
Fresh (N = 22)	335±75^a^	68±16^a^	79±13^a^	5.0±1.2^a^	44±11^a^
DMSO (N = 6)	65±36^b^	19±11^b^	36±9^b^	2.9±0.7^b^	38±7^a^
DMSO+AFP I (N = 8)	72±45^b^	29±10^b^	36±8^b^	3.8±0.5^bc^	39±10^a^
DMSO+AFPIII (N = 8)	76±62^b^	41±11^c^	54±11^c^	4.1±0.3^c^	41±5^a^

Values are expressed as means ± SD for % motility, motility duration and % vitality and as means ± SEM for VCL and VSL. Within each column, values with different superscript letters indicate significant statistical differences (*P*<0.05).

VSL: Velocity Straight Line.

VCL: curvilinear velocity.

Fertilization trials were performed using fresh and DMSO-AFPIII cryopreserved gametes (since AFPIII as cryoprotectant gave the best results in terms of sperm quality and proteins protection after cryopreservation). Our results demonstrated that there were no differences between fertilization ability obtained with fresh (77±9%) and cryopreserved spermatozoa (73±7%).

### Effect of antifreeze proteins on 2DE protein profiles of Isolated flagella and head plasma membranes from sea bream spermatozoa

**Figure 1:**
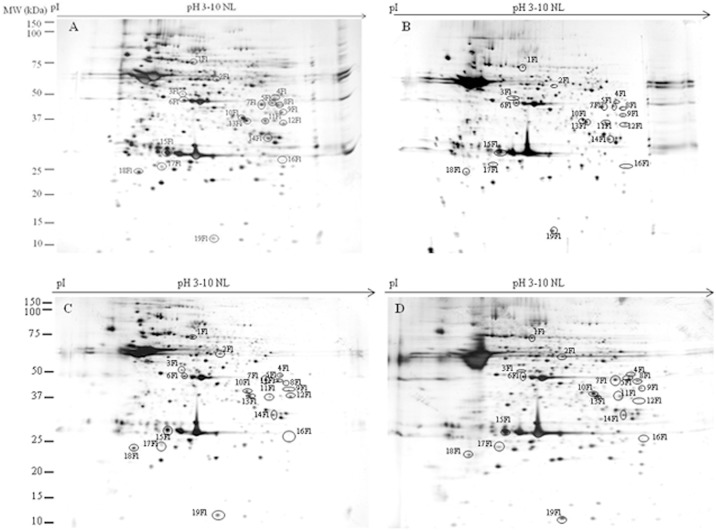
Representative 2-D gels of proteins extracted from flagella isolated from fresh (A), cryopreserved with DMSO (B), with DMSO plus AFPI (C) and with DMSO plus AFPIII (D) gilthead sea bream spermatozoa. Extracted flagellar proteins (60 µg) were loaded onto non-linear IPG strips pH 3–10, and following isoelectric focusing were separated by 12.5% SDS–PAGE. Gels were silver stained as described in Materials and Methods. Spots that changed their expression after cryopreservation procedure are highlighted with a circle.

To evaluate the ability of DMSO, AFPI and AFPIII to protect plasma membrane proteins during the freezing-thawing procedure, we compared the 2DE-profiles of proteins extracted from isolated flagella and heads of spermatozoa before and after the freezing-thawing (in DMSO or DMSO+AFPI or DMSO+AFPIII) procedure. Representative 2DE gels of proteins extracted from isolated flagella and plasma membrane heads of fresh and cryopreserved semen are shown in Figures 1 (flagella) and 2 (head plasma membranes).

The 2D experiments revealed 270 protein spots in gels obtained by using proteins extracted from isolated flagella and 150 spots in gels prepared with proteins extracted from head plasma membranes. In both cases the protein molecular masses ranged between 10 and 150 kDa and the isoelectric points ranged between 3.0 and 10 pH units.

**Figure 2:**
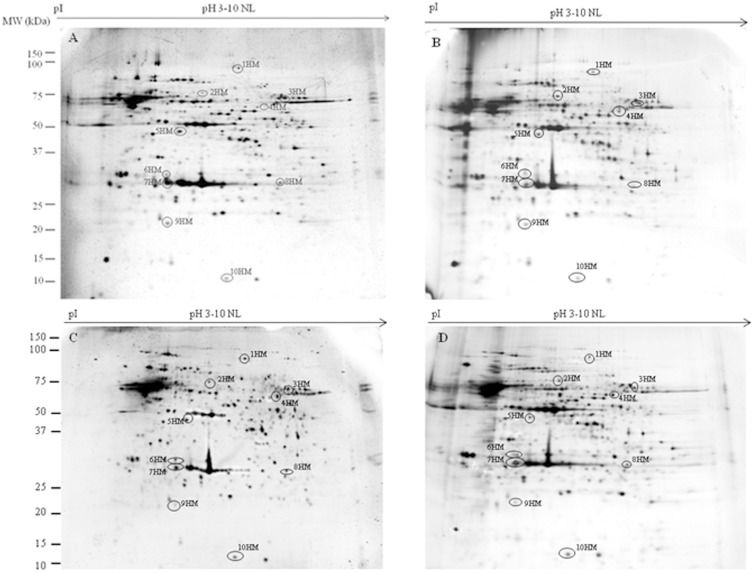
Representative 2-D gels of proteins extracted from the plasma membrane head isolated from fresh (A), cryopreserved with DMSO (B), with DMSO plus AFPI (C) and with DMSO plus AFPIII (D) gilthead sea bream spermatozoa. Extracted flagellar proteins (60 µg) were loaded onto non-linear IPG strips pH 3–10, and following isoelectric focusing were separated by 12.5% SDS–PAGE. Gels were silver stained as described in Materials and Methods. Spots that changed their expression after cryopreservation procedure are highlighted with a circle.

**Figure 3:**
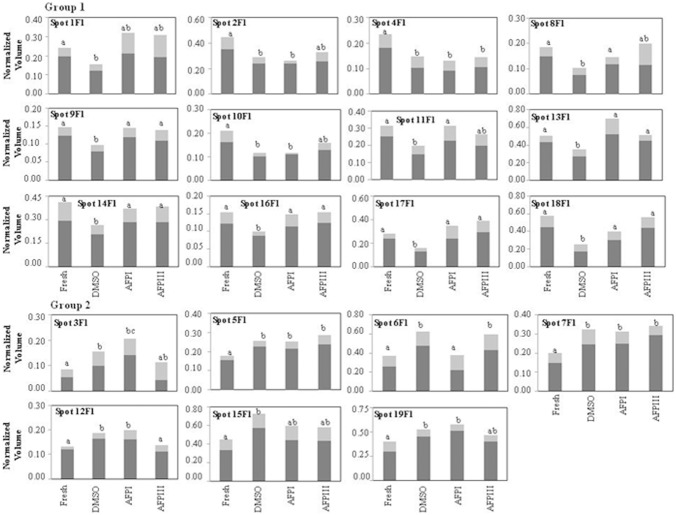
Differences in relative abundance of 19 flagella spots in fresh and cryopreserved sea bream spermatozoa. Spots are highlighted in Figure 1 and the *x* axis identified the experimental conditions (fresh semen or cryopreserved with DMSO, with DMSO plus AFPI and with DMSO plus AFPIII). The *y*-axis corresponds to the intensity of spots expressed as normalized spot volume. Spot intensity is expressed as mean (dark bars) ± SD (light grey bars) of normalized spot volume (N = 18, that correspond to 6 different fish and 3 replicates for each). Different letters in the same graph indicate that mean values are significantly different by Mann–Whitney test (p<0.05). The spots showing a decrease of their expression after cryopreservation procedure are reported in the group 1, and those showing an increase are reported in the group 2.

**Figure 4:**
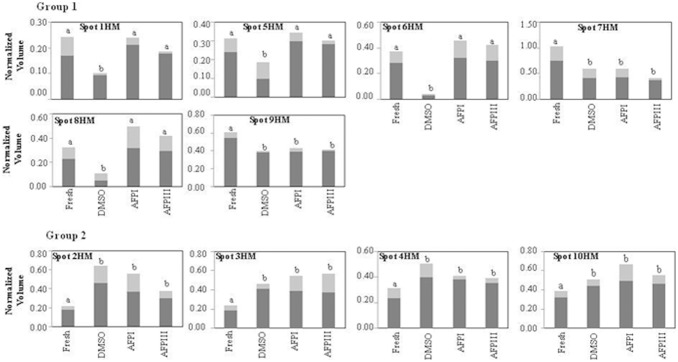
Differences in relative abundance of 10 sperm head membrane spots in fresh and cryopreserved sea bream spermatozoa. Spots are highlighted in Figure 2 and the *x* axis identified the experimental conditions (fresh semen or cryopreserved with DMSO, with DMSO plus AFPI and with DMSO plus AFPIII). The *y*-axis corresponds to the intensity of spots expressed as normalized spot volume. Spot intensity is expressed as mean (dark bars) ± SD (light grey bars) of normalized spot volume (N = 18, that correspond to 6 different fish and 3 replicates for each). Different letters in the same graph indicate that mean values are significantly different by Mann–Whitney test (p<0.05). The spots showing a decrease of their expression after cryopreservation procedure are reported in the group 1, and those showing an increase are reported in the group 2.

Quantification and statistical analysis using Mann–Whitney test (significance defined as *p*<0.05) showed that the normalized volume of 19 out of 270 protein spots obtained from flagella and 10 out of 150 protein spots extracted from sperm heads plasma membranes were ≥20% significantly different between at least one of the 3 cryopreserved groups and the fresh sperm. The gel location of these spots in sperm flagella and head plasma membranes is shown in Figures 1 and 2, respectively, while the corresponding normalized volumes, under the different experimental conditions, are reported in Figures 3 and 4. Following the freezing-thawing procedure, among the 19 highlighted spots from the flagella showed in Figure 1, 12 showed a significant decrease (group 1 of Figure 3) while 7 showed a significant increase (group 2 of Figure 3). The results demonstrate that DMSO, DMSO+AFPI and DMSO+AFPIII act differently on the expression of each of these spots. The use of DMSO as cryoprotectant in the absence of AFPs determined the decrease of all spots of group 1 and the increase of all spots of group 2. In the presence of AFPI (1 µg/ml) the freezing-thawing procedure determined the significant decrease of 3 out of 12 spots within the group 1 and the increase of 5 out of 7 within the group 2. Noteworthy, the addition of AFPIII (1 µg/ml) to DMSO extender determined the significant decrease of only 1 spot belonging to group 1 and the increase of only 3 spots within group 2.

The results shown in Figure 4 demonstrate that the freezing-thawing procedure differently affects the expression of protein spots in the head membrane when different cryoprotectants were added to the extender medium. The cryopreservation protocol carried out in DMSO extender significantly decreased 6 spots of group 1 and increased 4 out of 4 spots of group 2. In the presence of AFPI or AFPIII only 2 out of 6 spots were significantly decreased and 4 out of 4 were increased.

### Identification of proteins

Within the 29 protein spots (19 from flagella and 10 from head plasma membranes) which changed their expression following the freezing-thawing procedure, 18 (13 from flagella and 5 from head plasma membranes) were successfully detected by silver staining on 2DE preparative gels. Next, the detected spots were subjected to nanoHPLC ESI-Q-Tof MS/MS analysis for identification. Table 3 reports the results of such analyses with the identified 6 proteins clustered according to their putative function or their involvement in common metabolic pathways. 4 spots (7F1: alcohol dehydrogenase III; 10F1: glyceraldehyde-3-phosphate dehydrogenase; 11F1: cytosolic malate dehydrogenase and 14F1: axonemal dyenein light intermediate polyopeptide 1) were identified in flagella. The spots 15Fl (from flagella) and 7HM (from head plasma membranes) were both identified as carbonic anhydrase while spots 19F1 (from flagella) and 10HM (from head plasma membranes) were identified as superoxide dismutase [Cu-Zn].

**Table 3:** Identification by mass spectrometry of proteins that change their expression following the freezing thawing procedure.

SpotNumber	Proteinname	Acc.No.^a^	Species	MascotScore^b^	Uniquepetides^c^	Peptidesequence^d^	% SC^e^	pI^f^	MW^g^	pI^h^	MW^i^
7Fl	Alcohol dehydrogenase class-3	gi|5902742	*Sparus aurata*	78	2	ITQGQGLLPDK	5	6.71	40930	6.41	43000
						LVEDYMSK					
10Fl	Glyceraldehyde 3-phosphatedehydrogenase, testis-specific	gi|15146358	*Pagrus major*	463	8	VVAINDPFIDLK	32	6.36	36381	6.22	37000
						YHGEVSEEDGK					
						VVVSAPSPDAPMFVMGVNEEK					
						GAHQNIIPASTGAAK					
						LTGMAFR					
						VPVADVSVVDLTCR					
						LISWYDNEFGYSHR					
11Fl	cytosolic malate dehydrogenasethermolabile form	gi|14583131	*Sphyraena idiastes*	114	2	VLVVGNPANTNCLIAAK	8	6.60	36463	6.37	36500
						MDATAAELIEER					
14Fl	Axonemal dynein light intermediatepolypeptide 1	gi|30172570	*Mus musculus*	42	1	ELYSQCFDELIR	4	8.21	29947	6.46	31000
15Fl = 7HM	Carbonic anhydrase	gi|213514954	*Salmo salar* *Oncorhynchus mykiss.*	145	3	QFHFHWGASDDR FPCELHLVHWNTKIGAANPR	12	7.6	28823	5.48	28000
		gi|185135824									
19Fl = 10HM	Superoxide dismutase [Cu-Zn]	gi|27462182	*Pagrus major*	131	1	HVGDLGNVTAGADNVAK	11	5.7	16167	6.00	13000

a NCBInr. accession number.

b Mascot score obtained from MS/MS ion search against NCBInr [the Mascot score for an MS/MS match is based on the absolute probability (P) that the observed match between the experimental data and the database sequence is a random event, the reported score is − 10 Log(P) and the significance threshold is p<0.05; all the ion scores are higher than the threshold values (see also www.matrixscience.com).

c Number of unique identifed peptides.

d Amino acid sequences of identified peptides.

e Sequence coverage of the identified protein.

f Theoretical p*I.*

g Theoretical MW.

h Experimental p*I.*

i Experimental MW.

## Discussion

In the present study we demonstrate that the addition of AFPIII (1 µg/ml) to the extender medium significantly increased, with respect to control (DMSO extender), viability, motility rate and VSL of thawed spermatozoa. Moreover, we analysed, for the first time in sperm, the effect of AFPs on the profile of proteins extracted from isolated flagella and heads plasma membranes from sea bream spermatozoa. The results of the 2DE experiments suggest a higher ability of AFPIII, as compared to AFPI, to protect proteins during the freezing-thawing procedure. Studies of three-dimensional structures of both AFPs revealed important differences between them. AFPI is a small hydrophobic α-helix, whose direct interaction with phospholipids was suggested by Inglis *et al.* [27] working with liposomes. AFPIII is a globular protein with several hydrophilic and hydrophobic domains [37]. A direct interaction between AFPIII and the plasma membrane lipids has been indicated in our recent study [5]. In sea bream spermatozoa we showed that the addition of AFPIII to DMSO may avoid changes, which usually occur by freezing with DMSO alone, in membrane phospholipids composition as well as in the saturation/unsaturation degree of their component fatty acids. Activities and expression of several proteins are well known to be affected by the lipid micro domain surrounding membrane proteins [38]. This aspect should be taken into account when examining the protein expression of sperm cryopreserved in the different experimental conditions tested in the present study, i.e. in DMSO without or with AFPs. In addition, the higher ability of AFPIII (with respect to AFPI) to protect spermatozoa during the freezing-thawing procedure could be the result of the above reported mechanism or/and to a better sinergy between AFPIII and DMSO (with respect to the sinergy between AFPI and DMSO).

The observed decrease in protein abundance may be due either to degradation following freezing-thawing stress [12], or leakage of proteins from spermatozoa to the extracellular medium, as reported in human [34], boar [32] and bull sperm [33], while the observed increase of some protein spots could be due to one or more post-translation modifications (phosphorylation, acetylation, glycation, etc.) following the cryopreservation procedure, as we demonstrated in gilthead sea bream spermatozoa [39] or be a consequence of the freezing/thawing procedure and/or exposure to cryoprotectants on the regulation of mRNA translation, since, as it has been demonstrated in mammals, spermatozoa are not transcriptionally and translationally dormant cells [40]–[42].

In the present study the protein profiles were obtained by using both isolated flagella (Fig. 1) and head plasma membranes (Fig. 2) of sea bream spermatozoa. In our previous paper [12] the protein profile was obtained by using proteins extracted from the whole spermatozoa of sea bass. Due to the different starting samples (spermatozoa obtained from different fish species; whole spermatozoa and isolated head plasma membranes and flagella) and also to differences in sample preparation (amount of DMSO used, incubation time, isolation procedure, etc.) it is very difficult to compare the effect of the cryopreservation procedure on the detected protein markers obtained in the present and in our previous work.

Among the six identified proteins spot 7Fl was an Alcohol dehydrogenase class-III (ADH III). Spot 10Fl was identified as Glyceraldehyde 3-phosphate dehydrogenase (GAPDH). This protein is expressed in sperm at specific stages of spermiogenesis and can still be detected in mature spermatozoa of vertebrates [43]. Variations in the expression of GAPDH block the progressive motility of spermatozoa [44]. Thus, it can be hypothesized that the observed decrease of sperm motility after the freezing-thawing procedure (Table 2) could be attributed, at least partially, to the reduction of GAPDH expression. Protein spot 11Fl matched with the cytosolic malate dehydrogenase thermolabile form (MDH), which was previously found in the mid-piece of ram, boar and buffalo spermatozoa [45]. Interestingly, ADHIII, GAPDH and MDH show as common feature to be linked to the bioenergetic system of the cell. NADH+H^+^ is a product of both the ADHIII and GAPDH activities. In mammalian spermatozoa the transfer of reduced equivalents from the cytosol to the mitochondria occurs by the Malate-Aspartate shuttle, in which two isoforms of MDH, cytosolic and mitochondrial, are operative. By this shuttle, the hydrogen ions of the cofactor NADH produced in the cytosol can reach the electron transport chain in the mitochondria, and generate ATP by the oxydative phosphorylation (OXPHOS) system. Note that motility of fresh spermatozoa mainly depends on sperm ATP synthesized by mitochondrial OXPHOS [46]. Therefore, the observed reduction in GAPDH and MDH expression in cryopreserved sperm (in the presence of DMSO) may contribute to the reduced sperm motility observed after freezing-thawing procedure.

Spot 14Fl was identified as axonemal dynein light intermediate polypeptide 1 that belongs to the inner dynein arm light chain family and may play a dynamic role in flagellar motility [47].

The protein spots 15Fl-7HM matched with carbonic anhydrase (CA). In flatfish species CA plays an important role in the regulation of sperm motility [48] but in *S. aurata*, to the best of our knowledge, its role is unknown.

The spots protein 19Fl-10HM matched with superoxide dismutase [Cu-Zn] (CuZn-SOD). Oxygen free radicals have been implicated in a variety of circumstances relevant to the function of mammal spermatozoa; they may exert both toxic actions and participate in physiological processes [49]. It has been demonstrated that human spermatozoa are exceptionally well equipped with CuZn-SOD [49], and a relationship between loss of motility and peroxidation of spermatozoa lipids has been evidenced [50], [51]. The increase in CuZn-SOD we observed (at the flagella and heads level) could be a consequence of oxidative stress during the cryopreservation procedure.

The present study represents the first evidence in fish on the ability of AFPs to affect the protein expression of two different spermatozoa domains, flagella and the plasma membranes. The addition of AFPIII to the cryopreservation medium was particularly active in increasing the quality of the thawed spermatozoa. Our results are complementary to those of a previous study on the effects AFPs on membrane lipid composition [5], and demonstrated that during the freezing–thawing procedure, AFPs – mainly AFPIII - act by exerting a stabilizing effect on *S. aurata* sperm conservation by reducing the cryopreservation-induced changes in the protein expression pattern.

These results may prove useful in elucidating the molecular mechanism underlying alterations associated with sperm cryopreservation, and may have implications in the development of sperm conservation strategies.
